# Analysis of repetitive DNA distribution patterns in the *Tribolium castaneum *genome

**DOI:** 10.1186/gb-2008-9-3-r61

**Published:** 2008-03-26

**Authors:** Suzhi Wang, Marcé D Lorenzen, Richard W Beeman, Susan J Brown

**Affiliations:** 1 Department of Biology, Kansas State University, Manhattan, KS 66506, USA; 2 Grain Marketing and Production Research Center, Agricultural Research Service, United States Department of Agriculture, College Avenue, Manhattan, KS 66502, USA

## Abstract

**Background:**

Insect genomes vary widely in size, a large fraction of which is often devoted to repetitive DNA. Re-association kinetics indicate that up to 42% of the genome of the red flour beetle, *Tribolium castaneum*, is repetitive. Analysis of the abundance and distribution of repetitive DNA in the recently sequenced genome of *T. castaneum *is important for understanding the structure and function of its genome.

**Results:**

Using TRF, TEpipe and RepeatScout we found that approximately 30% of the *T. castaneum *assembled genome is composed of repetitive DNA. Of this, 17% is found in tandem arrays and the remaining 83% is dispersed, including transposable elements, which in themselves constitute 5-6% of the genome. RepeatScout identified 31 highly repetitive DNA elements with repeat units longer than 100 bp, which constitute 7% of the genome; 65% of these highly repetitive elements and 74% of transposable elements accumulate in regions representing 40% of the assembled genome that is anchored to chromosomes. These regions tend to occur near one end of each chromosome, similar to previously described blocks of pericentric heterochromatin. They contain fewer genes with longer introns, and often correspond with regions of low recombination in the genetic map.

**Conclusion:**

Our study found that transposable elements and other repetitive DNA accumulate in certain regions in the assembled *T. castaneum *genome. Several lines of evidence suggest these regions are derived from the large blocks of pericentric heterochromatin in *T. castaneum *chromosomes.

## Abstract 2

Approximately 30% of the *Tribolium castaneum* genome is comprised of repetitive DNA. These repeats accumulate in certain regions in the assembled *T. castaneum* genome, these regions might be derived from the large blocks of pericentric heterochromatin in *Tribolium* chromosomes.

## Background

The genome of the red flour beetle, *Tribolium castaneum*, has recently been sequenced and is currently being annotated. *Tribolium *has enjoyed a long history as a model for population genetics, and the recent development of genetic and genomic tools has contributed to its current status as a powerful genetic model organism for studies in pest biology as well as comparative studies in developmental biology [1]. In addition, as the first coleopteran genome to be sequenced, it will provide insight into the genomics of the largest metazoan order known.

Scaffolds containing approximately 90% of the genome sequence have been anchored to the ten chromosomes (*Tribolium *Genome Sequencing Consortium) in the molecular recombination map [2]. Understanding the structure and organization of this genome is the next major task. Automated analyses have been used to identify coding regions and to predict more than 16,000 gene models. In contrast, the much larger, non-coding part of the genome is more difficult to analyze, a situation that is exacerbated by the presence of considerable amounts of repetitive DNA. Although the role of repetitive DNA is not always clear, it has been implicated in gene regulation [3], disease-associated gene mutation [4] and genome evolution [5,6]. Understanding the abundance and distribution of repetitive DNA in *Tribolium *is required to understand the structure and function of the genome. In addition, once identified, different types of repetitive DNA can be masked to improve the quality of other homology-based searches.

Estimates of the repetitive DNA content in insect genomes vary widely. For example, reassociation kinetics indicate only 8-10% of the honey bee (*Apis mellifera*) genome and up to 24% of the *Drosophila melanogaster *genome are composed of repetitive DNA [7,8], while the repetitive DNA content in the *Tribolium *genome appears to be over 42% [9,10], nearly the level observed in the human genome [11]. In light of this estimate, we might expect to find repetitive DNA elements that are highly dispersed throughout the *Tribolium *genome, such as transposable elements, as well as those clustered in tandem arrays, such as microsatellites (repeat units of 1-6 bp), minisatellites (7-100 bp) and satellites (>100 bp).

Whether highly dispersed or tandemly repeated, repetitive DNA is not randomly distributed throughout a genome. Heterochromatic regions near centromeres and telomeres are often rich in repetitive sequences, including transposable elements and satellites. Heterochromatin is distinguished from euchromatin by its molecular and genetic properties, such as DNA sequence composition, high levels of condensation throughout the cell cycle [12], low rates of meiotic recombination [13] and the ability to silence gene expression [14]. Most eukaryotic genomes include a significant fraction of heterochromatin. In insects, large blocks of pericentric heterochromatin have been identified by C-banding. In tenebrionid beetles, including *Tribolium*, large blocks of pericentric heterochromatin often constitute 25-58% of the genome [15]. C-banding in *Tribolium *species has revealed large blocks of pericentric heterochromatin. For example, 40-45% of the *Tribolium confusum *genome consists of pericentric heterochromatin [16] and pericentric heterochromatin has been characterized by *Hpa*II-banding in *T. castaneum *[17]. The highly repetitive nature of heterochromatic DNA makes it refractory to cloning, sequencing and subsequent assembly, resulting in its under-representation in genome sequencing projects. Indeed, special efforts had to be directed towards analysis of heterochromatin in *Drosophila *[18].

We used three complementary approaches to identify repetitive DNA in the newly assembled *T. castaneum *genome. Specifically, we used Tandem Repeat Finder (TRF) [19] to find tandem arrays of repetitive DNA, TEpipe [20] to identify transposable elements based on structural features and sequence conservation, and RepeatScout [21] for *de novo *identification of repeat families in large, newly sequenced genomes such as that of *Tribolium*, for which hand-curated repeat databases are not available. We then used RepeatMasker (version open-3.1.0, RepBase Update 10.05) [22] with these newly compiled repeat sequence libraries to find homologous copies and determine the abundance and distribution of repetitive DNA in the *Tribolium *genome. Not surprisingly, over 50% of the unmapped DNA sequence consists of repetitive DNA. However, we were surprised to find that within the scaffolds included in the chromosomes, repetitive DNA accumulates in patterns resembling the large blocks of pericentric heterochromatin previously identified in *Tribolium *[17]. Analyses of gene content, intron size, and recombination rates across the genome provide additional evidence for the identification of putative heterochromatic versus euchromatic regions, and suggest that the *T. castaneum *genome sequence assembly and scaffold mapping efforts successfully captured not only the euchromatin, but a significant fraction of the heterochromatic DNA as well.

## Results and discussion

The *T. castaneum *genome was recently sequenced at seven-fold redundancy, and a draft assembly produced (*Tribolium *Genome Sequencing Consortium). The assembled genome, which is approximately 151 Mb in size, consists of 481 scaffolds and 1,849 additional contigs and reptigs that failed to assemble into scaffolds using automated methods. In the second version of the *Tribolium *genome assembly, release Tcas_2.0, 140 of these scaffolds (representing 70% of sequenced genome) were anchored to 10 chromosomes (9 autosomal chromosomes and the X) that were previously constructed by high-resolution recombinational mapping using bacterial artificial chromosome and expressed sequence tag markers [2]. These scaffolds were assembled into ten 'chromosomes' (CH1-CH10) based on the order and orientation of the mapped marker sequences; 300 kb spacer sequences (Ns) were inserted to delineate the individual scaffolds. The remaining scaffolds, contigs and reptigs were concatenated into a single chimeric chromosome designated 'unknown'. Since the genetic map does not include the Y chromosome, scaffolds belonging to the Y must be contained within the 'unknown' file. Before beginning our analysis, we assessed the accuracy of each chromosome build by verifying the location of each marker. Several discrepancies were uncovered and corrected: four misassigned scaffolds were moved from one end of CH1(X) to their correct location at one end of CH2; the orientation of two scaffolds in CH7 were reversed; two misassigned scaffolds were moved from CH5 to their correct locations on CH1 and CH7; and another misassigned scaffold was moved from CH6 to CH8. In addition, 23 newly mapped scaffolds were added to CH1(X), CH2, CH3, CH5, CH7, CH8, CH9 and CH10, increasing the portion of the anchored genome to 86.5%.

### Characterization of tandem repetitive DNA

We used TRF to survey the assembled *Tribolium *genome for arrays of tandem repeats. To validate our results, we performed a similar survey of the *D. melanogaster *genome using the same parameters, and were encouraged in that our results compare favorably with those previously reported for this insect [23,24]. Mononucleotide repeats (≥15 tandem copies), dinucleotide repeats (≥7 copies) and trinucleotide repeats (≥5 copies) were considered, as well as tetra-, penta- and hexanucleotide repeats (≥4 copies) and longer satellites (≥2 copies). Sequence identity greater than 80% between repeats within an array was required. Using these parameters, we found that microsatellites (1-6 nucleotides per repeat unit) are less abundant in *Tribolium *than in *Drosophila *(Table 1). Similarly, minisatellites (between 7 and 100 nucleotides) are slightly less abundant in *Tribolium*. However, satellites over 100 nucleotides, which are quite rare in *Drosophila*, are prevalent in *Tribolium*. The total amount of tandem repetitive DNA in kilobases is comparable in the two insects but, due to the somewhat larger genome, the average density of tandem repeat loci in *Tribolium *is actually lower than in *Drosophila*.

**Table 1:** Abundance and average density of microsatellites, minisatellites and satellites in the *D. melanogaster *and *T. castaneum *genomes identified by TRF

	Number of base pairs	Percentage of genome	Number of loci	Average density* (loci/Mb)
***Tribolium***				
Microsatellites	591,105	0.4	17,328	114
Minisatellites	3,112,304	2.1	120,474	796
Satellites	3,775,523	2.5	4,272	28
Total tandem repeats	7,478,923	4.9	142,074	939
Genome	151,333,735			
				
***Drosophila***				
Microsatellites	1,442,241	1.0	52,906	367
Minisatellites	3,590,753	2.5	126,237	876
Satellites	1,075,701	0.7	1,343	9
Total tandem repeats	6,108,695	4.2	180,486	1,253
Genome	143,955,363^†^			

*For the *Tribolium *genome, average density = number of repeats/151 Mb; for the *Drosophila *genome, average density = number of repeats/144 Mb. ^†^The size of the *Drosophila *genome was calculated by summing the euchromatin (124,006,872 bp) and heterochromatin (19,948,491 bp) not including sequence gaps.

**Figure 1:**
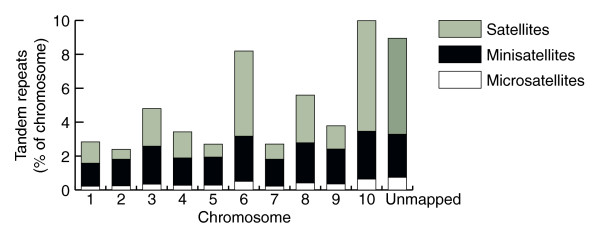
Distribution of microsatellites, minisatellites and satellites on each chromosome of the *T. castaneum *genome.

In *Tribolium*, micro- and minisatellites are evenly distributed between chromosomes, including the concatenated group of unmapped scaffolds, but certain chromosomes contain more long satellites (>100 bp) than others (Figure 1). Such variability may reflect real differences in the organizational structure of each chromosome or it might simply be an artifact caused by the assembly status of the genome, especially in light of the large number of scaffolds containing long satellites that lack chromosome assignments.

**Figure 2:**
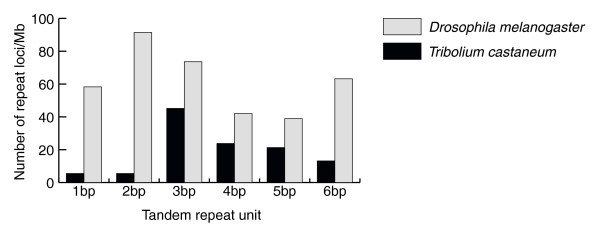
Frequencies of microsatellites per million base pairs in the *D. melanogaster *and *T. castaneum *genomes.

Trinucleotides are the most abundant type of microsatellite in *Tribolium*, while mono- and dinucleotide repeats are comparatively rare (Figure 2). In contrast, dinucleotides predominate in *Drosophila*. In *Tribolium*, microsatellite repeats of all lengths are A/T-rich, while C/G-rich repeats are rare, which may explain the limited success of previous attempts to generate DNA libraries enriched in microsatellite sequences [25]. The GC content in the *Tribolium *genome is 34%, while in *Drosophila *it approaches 41%. This may, at least in part, account for the fact that A/T-rich repeats are considerably more plentiful than G/C-rich repeats in *Tribolium*.

Results similar to ours have been reported both for *Tribolium *[26,27] and *Drosophila *[24]. Comparison of these studies reveal small differences in the total number of microsatellites identified, but the overall profile of microsatellite content is consistent between studies despite the differences in software, parameters, and genome files used to define and identify the microsatellites. In each study, microsatellites composed of dinucleotide repeats predominate in *Drosophila*, while trinucleotide repeats are more abundant in *Tribolium*.

### Distribution of transposable elements in the *Tribolium *genome

Transposable elements (TEs) are an abundant component of most, if not all, eukaryotic genomes. For example, TEs have been estimated to make up about 3.7% of euchromatin and 15.1% of heterochromatin in the *Drosophila *genome [28], and, in the recently assembled *Anopheles gambiae *genome, TEs constitute about 16% of the euchromatin and more than 60% of the heterochromatin [29]. TEs are divided into two classes, depending upon whether their transposition is RNA-mediated or DNA-mediated. DNA-mediated transposons are mobilized by direct replication of the DNA. RNA-mediated retrotransposons are mobilized by reverse transcription, and encode reverse transcriptase. Reverse transcriptase-encoding TEs include long terminal repeat (LTR) retrotransposons and non-LTR retrotransposons, which have no terminal repeats. In homology searches using TEpipe to identify TEs in the *T. castaneum *genome assembly (S Wang, Z Tu, J Biedler and S Brown, unpublished), we found representatives of 69 families of non-LTR retrotransposons, 48 families of LTR retrotransposons and 45 DNA transposon families. In the present study, we have determined the percent of the assembled genome occupied by each type of TE (Table 2). The DNA transposon library is smaller (78.6 Mb) than the non-LTR (238.1 Mb) and LTR (290.2 Mb) libraries. However, DNA transposons occupy a slightly larger percentage of the genome (2.2%), which is consistent with the higher average copy number of DNA transposons (Table 2). Altogether, TEs constitute 5.9% of the assembled genome.

**Table 2:** Summary of LTR and non-LTR retrotransposons and DNA transposons identified by TEpipe in the *T. castaneum *genome assembly

Class	TE library* (kb)	Number of families	Percentage of genome^†^	TE length range (bp)	Average length (bp)	Copy number (range)	Average copy number	GC content range (%)	Average GC content (%)
Non-LTR	238.1	69	2.0	786-6,820	3,363	1-2,556	161	27.15-57.94	38.14
LTR	290.2	48	1.7	3,292-11,097	6,019	1-1,634	202	30.61-53.21	39.31
DNA transposons	78.6	45	2.2	456-4,878	1,746	1-8,949	420	30.90-46.08	37.22

*Non-LTR, LTR and DNA transposon TE libraries were produced by TEpipe, which is based on sequence similarity searches using conserved domains from reverse transcriptase and transposase. ^†^To calculate the abundance of TEs in the *Tribolium *genome assembly, RepeatMasker was run using our TEpipe libraries.

The total density of TEs per chromosomes varies (Additional data file 1), and is higher on CH3, CH6, CH8, CH9 and CH10 than on the others. Even when the density of non-LTR, LTR and DNA transposons on each chromosome was analyzed separately, a higher density of each type was observed on these chromosomes than on the others. As stated previously with respect to the distribution of microsatellites, these differences may indicate true differences in the organizational structure of these chromosomes, or they may merely reflect the still-incomplete state of the assembly and map of the genome sequence. A very high density is found in the unmapped scaffolds, contigs and reptigs (Additional data file 1), suggesting that TEs are often located in genomic regions that are difficult to assemble.

### *De novo *identification of repetitive DNA in the *T. castaneum *genome

To determine whether the *Tribolium *genome contains additional repetitive DNA, perhaps not found by TRF or TEpipe, we used RepeatScout to search *de novo *for repeats. TE databases such as Repbase Update [30] contain libraries of repetitive elements that have been compiled for well-studied genomes, for example, *D. melanogaster*, *Homo sapiens*, *A. gambiae *and others. Prior to our study, only a few repetitive elements had been studied in *Tribolium*, including a 360 bp satellite [31] and a gypsy-class retrotransposon named Woot [10]. Little is known about the overall profile of repetitive DNA in this genome. The RepeatScout algorithm employs Nseg [32] and TRF [19] to remove low-complexity repeats and tandem repetitive DNA, respectively. For well-studied genomes, RepeatScout uses GFF files describing exon locations to remove repeat families containing protein encoding open reading frames. Since similar files are not available for newly sequenced genomes such as that of *Tribolium*, we used BLASTX to identify repeats that produce significant matches to known proteins in UniProt (release 6.0) [33], which were subsequently removed. To retain putative TEs in the RepeatScout library, matches with reverse transcriptases and transposases were not removed. The library of repetitive elements found by RepeatScout masked almost 25% of the genome, which is significantly more than the TRF (4.5%) or TEPipe (5.8%) libraries, and suggests that there are additional novel repetitive sequences in the *Tribolium *genome.

Before analyzing the resulting *Tribolium *repeat library, we generated a RepeatScout library for *Drosophila *using the same default parameters. Then we used RepeatMasker to compare our *Drosophila *RepeatScout library with the existing *Drosophila *Repbase library (release 10.05) [30]. The RepeatScout library masked 84% of the Repbase library, while the Repbase library masked 64% of the RepeatScout library (data not shown). These results indicate that RepeatScout identified a majority of known *Drosophila *transposon sequences, as well as other types of repetitive DNA, which might include previously unannotated transposons or highly repetitive satellites. These results encouraged us to analyze the *Tribolium *RepeatScout library in some detail.

The *Tribolium *RepeatScout library contains 4,475 repeat families with a total length of 1.41 Mb (Table 3 and Additional data file 2). Twenty-six percent of the 151 Mb *Tribolium *genome is composed of repeats found in this RepeatScout library (Table 3). In comparison, the *Drosophila *RepeatScout library contains 3,297 repeat families with a total length of 2.51 Mb. This constitutes 20% of the 144 Mb *Drosophila *genome. The *Drosophila *RepeatScout library contains fewer and longer repeats that mask a smaller percent of the *Drosophila *genome, while the *Tribolium *RepeatScout library contains more and shorter repeats that constitute a larger percent of the *Tribolium *genome. This difference may be due, in part, to the fact that 64% of the *Drosophila *RepeatScout library consists of known transposons, with an average length of 4 kb. To estimate the proportion of TE-derived sequences in the *Tribolium *RepeatScout library, the TEpipe libraries (described above) were used to mask the *Tribolium *RepeatScout library (Additional data file 3). We found that RepeatScout did not find all the TE sequences identified by TEpipe. This is probably due, at least in part, to the fact that TEpipe uses TBLASTN to identify DNA sequences encoding protein domains that are required for transposition and are highly conserved at the amino acid level but not necessarily at the DNA level. To be included in the RepeatScout library, an element must be highly conserved at the DNA level. In addition, to identify full length TE elements, the protein encoding fragments were extended by 1 kb or more in both directions. Transposable elements identified in this manner may not be repetitive in the genome or may be diverging at the DNA level as they degenerate. Thus, RepeatScout identified fewer sequences from TEs than did TEpipe. Indeed, when we compared the coverage of the conserved protein domains, 93% of the reverse transcriptases and 83% of the transposases in the TEpipe libraries were masked by RepeatScout. In contrast, when we used the TEpipe libraries to mask the RepeatScout library, we found that less than 30% of the RepeatScout library is derived from TEs (Table 4 and Additional data file 3). This is most likely due to that fact that RepeatScout identifies repetitive elements larger than 50 bp with at least three copies in the genome.

**Table 3:** Comparison of repetitive DNA in *D. melanogaster *and *T. castaneum *identified by RepeatScout

Genome	Assembled genome size (Mb)	RepeatScout library size (Mb)	Number of repeat families	Amount of genome (Mb)	Percentage of genome	GC content of library (%)	GC content of the genome (%)
*Drosophila*	144	2.51	3,297	29.3	20	59.94	41.44
*Tribolium*	151	1.41	4,475	38.9	26	34.52	33.87

**Table 4:** Analysis of the *Tribolium *repeat library produced by RepeatScout

Repeat class	Total repeat family length (kb)	Number of repeat families	Percentage of RepeatScout library	Percentage of genome*	Repeat family length range (bp)	Repeat family average length (bp)	Repeat family copy number range	Repeat family average copy number	Repeat family GC content range (%)	Repeat family average GC content (%)
HighA^†^	26.1	31	1.9	7.1	160-1,771	841	323-4,337	1,368	23.05-33.75	28.37
Mid^‡^	220.3	304	15.6	7.4	67-4,881	725	11-1,746	204	13.46-47.51	30.19
Low^§^	738.2	3,237	52.3	4.7	51-4,520	228	3-215	14	12.28-71.15	33.61
HighB^¶^	4.6	5	0.3	1.6	982-1,277	921	432-3,531	1,306	26.58-31.32	29.67
360 bp satellite^¥^	0.4	1	0.2	0.3	-	-	1,122	-	-	26.31
Transposable elements^#^	406.2	896	28.9	4.4	51-11,289	453.3	3-2,471	27	15.28-65.93	38.59

*RepeatMasker was used to determine the percent of the genome occupied by each repeat class. ^†^High repetitive A, 31 repeat sequences that each masked >0.1% of the genome. ^‡^Middle repetitive, 304 repeat sequences that each masked >0.01% and <0.1% of the genome. ^§^Low repetitive, 3,237 repeat sequences that each masked <0.01% of the genome. ^¶^High repetitive B, repeat sequences that each masked >0.1% of the genome, but show a different distribution pattern to the HighA repeat sequences. ^¥^360 bp satellite was removed from the HighA class for separate analysis. ^#^Transposable elements were removed from the HighA, Mid, and Low repetitive classes for separate analysis.

The majority of elements in the *Tribolium *RepeatScout library likely represent some type of satellite, since none of them encode proteins having significant BLAST and some are highly tandemly repeated in the genome. Furthermore, the GC content of the *Tribolium *RepeatScout library (34%; Table 3) is similar to that of the *Tribolium *genome and much lower than that of the *Drosophila *RepeatScout library (59.9%), indicating that repetitive sequences in *Tribolium *are likely to be AT-rich. In comparison, the average GC content of the TE identified in *Tribolium *is higher (Table 2), as expected for sequences that encode functional proteins.

In our analysis of the individual repeat families in the *Tribolium *RepeatScout library, we considered sequences from TEs (896) as a separate class. The remaining elements were categorized into High, Mid and Low repetitive classes based on the percent of the genome (in bp) that they occupy (Table 4 and Additional data file 4). The High repetitive class includes 36 repeat elements, each of which occupies more than 0.1% of the genome. Five of these highly repetitive sequences (designated the HighB class), are distributed in a pattern complementary to that of all the other highly repetitive sequences (designated the HighA class), as discussed in detail below. The Mid repetitive class includes 304 repeat elements, which each occupy between 0.01% and 0.1% of the genome. The Low repetitive class includes 3,237 repeat elements, which each constitute less than 0.01% of the genome.

Tandem arrays of one, highly repetitive 360 bp satellite have been estimated to constitute as much as 17% of the *Tribolium *genome [31]. This satellite was identified in the RepeatScout library and analyzed separately from the other classes (Table 4). In our analysis, the 360 bp satellite constitutes 0.3% of the assembled *T. castaneum *genome. Since these arrays may not assemble well, we looked for the 360 bp satellite in the bin0 sequences, which contains sequence reads that failed to assemble; 15% of the bin0 sequences match the 360 bp satellite with an E-value below 1e-05. Since the 400 Mb of sequence in bin0 is highly redundant, it was not possible to confirm how much of the genome is composed of this satellite, but our data do not contradict previous estimates.

**Figure 3:**
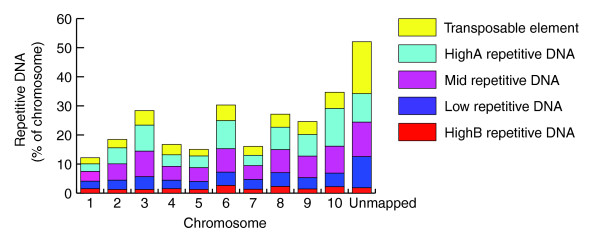
Distribution of repetitive elements and transposable elements identified by RepeatScout and TEpipe on the *Tribolium *chromosomes. Repeat elements in the RepeatScout library were classified into High, Mid and Low classes based on the percent of the genome (in bp) that they masked. High repetitive, 37 repeat sequences that each masked >0.1% of the genome. Middle repetitive, 352 repeat sequences that each masked >0.01% and <0.1% of the genome. Low repetitive, 3,179 repeat sequences that each masked <0.01% of the genome.

As previously noted for the TEs identified by TEpipe, the repetitive DNA sequences identified by RepeatScout are not uniformly distributed in the genome. Most chromosomes contain less than 20% repetitive DNA but CH3, CH6, CH8, CH9 and CH10 each contain more (Figure 3). The percentage of HighA, Mid and Low type repeats is higher in CH3, CH6, CH8, CH9 and CH10 than on the other chromosomes, while the percentage of HighB is higher only in CH6, CH8 and CH10. All five of these chromosomes contain more TE sequences identified by RepeatScout, as was also true of the results obtained using the TEpipe library. It is also important to note that more than 52% of the unmapped sequences are composed of repetitive DNA, again suggesting that it predominates in regions that are difficult to assemble into long scaffolds.

### Repetitive DNA library comparison provides an estimate of total repetitive DNA in the genome assembly

We compared the sequences in the libraries generated by these three methods to eliminate redundancy and to estimate the total amount of repetitive DNA in the *Tribolium *genome assembly (Table 5). The RepeatScout library has 124 sequences in common with the TRF library and 896 sequences in common with the TEPipe libraries. After removing the redundant sequences and applying RepeatMasker, about 30% of the *Tribolium *genome appears to be composed of repetitive DNA, but this estimate is likely to be conservative since a large amount of repetitive DNA was detected in bin0 (sequences that did not assemble).

**Table 5:** Estimated total repetitive DNA in *T. castaneum *genome assembly

Tools	Percentage of genome masked	Percentage of masked genome overlapping with RepeatScout
RepeatScout	25.7	N/A
TRF	4.9	1.5
TEpipe	5.8	5.2
Total	36.4	6.7
Total repetitive DNA in *Tribolium *genome	36.4 - 6.7 = 29.7	

### Distribution of repetitive DNA on each chromosome may identify regions derived from heterochromatin

**Figure 4:**
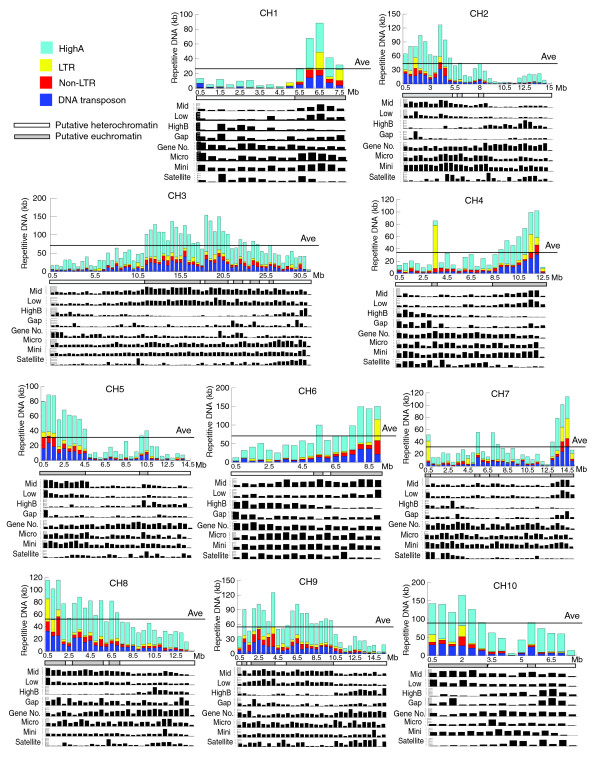
Density and distribution of repetitive DNA on each chromosome of *T. castaneum*. The total length (kb) of repetitive DNA in each 500 kb interval along the chromosome is plotted. The 300 kb placeholders were not included in the chromosomes. Sequencing gaps are included in the calculation if they are ≥50 bp. The length cutoff for parsing the RepeatMasker results was 50 bp. The HighA class includes the 360 bp satellite. Gene number, gap length and distribution of other repetitive classes within the 500 kb intervals are shown below the main graph for each chromosome. The combined average of HighA repeats and TE per 500 kb along the chromosome is depicted as a black line.

TEs and satellite DNA are known to accumulate in chromosomal regions that are composed largely of heterochromatin, as has been described for *D. melanogaster*, *H. sapiens*, *A. gambiae *and other species [12,16,34-38]. To determine whether the types of repetitive DNA identified in this study might show differential accumulation in the genome, we analyzed the distribution of repetitive DNA (length ≥50 bp) within 500 kb intervals (Figure 4) along the length of each as performed previously for 250 kb intervals in *D. melanogaster *[39]. The unmapped scaffolds were not included because they are not long enough to reliably analyze, thus reducing the size of the analyzed genome to 137.7 Mb. As shown in Figure 4, repetitive DNA is not uniformly distributed within each chromosome (similar results were obtained with 100 kb intervals; Additional data file 5). To characterize these distribution patterns, we compared the observed density of HighA class repeats and TEs within each interval to the average density expected if they were uniformly distributed. Since higher densities of repetitive DNA may correlate with heterochromatin, we considered intervals where the observed density/average density is significantly greater than one to be putative heterochromatin. Conversely, intervals where the observed density/average density is less than or equal to one were considered to be euchromatin (designated by open and closed boxes, respectively, below the graphs in Figure 4). With respect to this classification, it is important to note that most of the intervals in which the calculated ratios approach one are located at the boundaries of putative hetero- and euchromatin. In regions distant from these boundaries the ratio of observed to expected repetitive DNA is significantly greater than one (putative heterochromatin) or significantly lower (putative euchromatin) (*P *< 0.05). These criteria provide a basis for discussion here, but they are likely to be modified somewhat in future analyses that specifically target heterochromatic regions. By these criteria, 54.7 Mb out of the total 137.7 Mb of anchored sequences, or 40%, may be derived from heterochromatic regions (Additional data file 6). The amount of putative heterochromatin varies from one chromosome to the next; CH7 contains the least, while CH2, CH3, CH8, CH9 and CH10 contain the most. Half of CH9 and CH10 appear to be composed of putative heterochromatin. These results correlate well with the amount of repetitive DNA found in each CH, in that the CHs with more repetitive DNA overall also appear to have larger proportions of putative heterochromatin.

Some but not all of the other classes of repetitive DNA are distributed similar to the HighA repeats and TEs (Figure 4 and Table 6). The Mid and Low abundance classes of repetitive DNA indentified by RepeatScout are distributed in patterns similar to the HighA repeats and TEs. In contrast, the five elements in the HighB class are distributed in the opposite pattern along each chromosome. Micro- and minisatellites identified by TRF appear to be evenly distributed within the putative heterochromatic and euchromatic regions on each chromosome, while the longer, tandemly repeated satellites appear to accumulate in the same intervals as the HighB class repeats. These may represent the actual distributions, although the following caveat must be considered: if an element is highly repetitive, most of the copies may be either unassembled or not anchored in the chromosomes. When the longer satellites from the TRF library were compared to those in the RepeatScout library, 74% of the long tandemly repeated satellite elements were also found as monomers in the RepeatScout library. For example, 19 of the 31 repeats in the HighA class, which we have shown to accumulate in putative heterochromatin, are also found in the TRF libraries. The TRF results indicate that more short arrays of these satellites are found in the putative euchromatin than in heterochromatin in the current assembly. However, gaps in the genomic sequence (which occur more often in the putative heterochromatin than euchromatin) are often flanked by monomer or partial copies of these satellites. These sequencing gaps (Figure 4) are likely to represent regions of highly repetitive DNA that may not have been cloned or sequenced, or if sequenced, could not be assembled.

**Table 6:** The distribution of repetitive DNA in putative heterochromatin and euchromatin in assembled anchored genome of *T. castaneum*

Repeat element	Total length (kb)	Amount in heterochromatin (kb)	Amount in euchromatin (kb)	Percentage in heterochromatin	Percentage in euchromatin
Total anchored DNA	137,758	54,754	83,004	39.70	60.30
HighA	8,729	5,633	3,096	64.53	35.47
Mid	8,769	5,633	3,096	59.00	41.00
Low	4,915	2,893	2,022	58.86	41.14
HighB	2,045	267	1,778	13.06	86.94
Non-LTR	1,370	962	408	70.22	29.78
LTR	1,042	896	312	74.17	25.83
DNA transposon	2,579	1,963	616	76.11	23.89
Microsatellite	439	188	251	42.82	57.18
Minisatellite	2,593	1,152	1,441	44.43	55.57
Tandem satellites	2,621	646	1,975	24.65	75.35

We used nonparametric statistics to determine whether or not the distribution of these putative heterochromatic intervals along each chromosome is random. Intervals defined as putative heterochromatin by the above analysis were denoted by 1 and euchromatin by 0. The distribution of these intervals was analyzed using one-sample run tests [40,41]. We found that the intervals of putative heterochromatin and euchromatin are not randomly distributed on each chromosome (*P *< 0.05; Table 7). Heterochromatic intervals aggregate at one end, with the exception of the longest chromosome, CH3, where the intervals are grouped closer to the center. We compared the location of the putative heterochromatic regions on each chromosome (Table 7) with the location of pericentric heterochromatin blocks characterized by *Hpa*II-banding in *T. castaneum *[17]. In *Tribolium*, correlation between chromosomes and linkage groups in the recombination map is difficult at best. However, cytological studies indicate that the longest chromosome is centromeric, while the remaining chromosomes are much shorter and mostly telocentric. Interestingly, we found that the putative heterochromatic intervals are centrally located on CH3, the longest chromosome build in the genome sequencing project. The acrocentric X chromosome is the second longest, but the low scaffold density of this chromosome build in the sequencing project precludes analysis of heterochromatin localization. The remaining CHs in the assembled genome have fewer sequences anchored to them, and the putative heterochromatic intervals tend to accumulate at one end. Such striking similarity between the distribution pattern of repetitive DNA in the genome sequence and the *Hpa*II chromosome-banding patterns of pericentric heterchromatin supports the hypothesis that the regions accumulating repetitive DNA are likely derived from heterochromatin. Indeed, the 360 bp satellite, which is a member of the HighA class repeats, was previously shown to hybridize to the regions of pericentric heterochromatin visible in metaphase chromosomes [31].

**Table 7:** Nonparametric one-sample runs test for randomness of distribution of heterochromatin and euchromatin blocks

CH	*n*	*n1*	*n2*	*r*	Interval sequence*
CH1	15	5	10	2^†^	000000000011111
CH2	30	12	18	6^†^	111111111101000100000000000000
CH3	61	24	37	11^†^	0000000000000000000000111111111111101111110011011001000000000
CH4	25	8	17	5^†^	0000001000000000011111110
CH5	29	11	18	4^†^	11111111100000000001100000000
CH6	18	7	11	4^†^	000000000010111111
CH7	30	8	22	8^†^	100000000010011000000000011110
CH8	28	12	16	6^†^	1111011111101100000000000000
CH9	31	16	15	7^†^	0101111100111111111100000000000
CH10	15	7	8	4^†^	111111000010000

Columns: CH, chromosome; *n*, total interval; *n1*, the number of observations of 1; *n2*, the number of observations of 0; *r*, the total number of runs. *We calculated the average density of TEs and HighA satellites per 500 kb for each chromosome and then compared the observed density in each 500 kb interval across the chromosome to this average. If the observed density/average density is >1, this interval was considered to be putative heterochromatin and was denoted as 1. If the observed density/average density is ≤1, this interval was considered to be euchromatin and was denoted as 0. ^†^*P *< 0.05.

### Gene density in putative heterochromatin

Heterochromatin is known to be gene-poor in comparison to euchromatin [18,42-45]. Thus, we hypothesized that if the regions accumulating repetitive DNA are derived from heterochromatin, then they might contain fewer genes than the repetitive DNA-poor intervals. To test this hypothesis, the density of GLEAN gene models (Baylor HGSC, *Tribolium *Genome Project) in putative euchromatin was compared with that in the putative heterochromatic intervals (Table 8). Only the 14,511 genes predicted from the anchored sequences were used in this calculation. The density of genes within the intervals of the anchored genome defined as putative heterochromatin is significantly lower (83 genes/Mb) than in the rest of the mapped genome (120 genes/Mb) (chi-square test, *P *< 0.01; Table 8). The number of exons and introns per Mb in the putative heterochromatic regions (340/Mb and 339/Mb, respectively) are also reduced compared to that found in euchromatin (547/Mb and 543/Mb, respectively), consistent with the lower average gene density there (chi-square test, *P *< 0.01). Although the average exon size, average exon size/gene and average exon number/gene do not differ between these regions, the average intron size is larger in the heterochromatic regions (2,711 bp) than in euchromatin (1,705 bp), *P *< 0.01. These longer introns result in larger genes (6.5 kb) relative to those in euchromatin (5.0 kb). In summary, there are fewer genes in the putative heterochromatic regions than in euchromatin and they contain longer introns. These differences are likely due to an abundance of TEs and repetitive DNA not only in intergenic regions, but also in the introns of genes in the putative heterochromatin.

**Table 8:** Analysis of density, average size and GC content of genes, exons and introns in putative heterochromatin and euchromatin of *T. castaneum*

	Heterochromatin	Euchromatin	Average in anchored genome
Length (Mb)	54.7	83.0	-
Percentage in anchored scaffolds	40	60	100
GC content (%)	32.4	35.1	34.0
Average gene size (kb)	6.5	5.0	5.5
Gene* size/MB (kb)	546	602	579
Number of genes/Mb	83	120	105
Gene GC content (%)	33.6	36.5	35.4
Average exon size (bp)	312	329	314
Exon* size/gene (bp)	1,272	1,501	1,429
Number of exons/gene	4.1	4.6	4.4
Number of exons/Mb	340	547	465
Exon GC content (%)	44.8	46.3	45.9
Average intron size (bp)	2,711	1,705	1,999
Intron* size/gene (bp)	5,238	3,694	4,180
Number of introns/gene	3.1	3.6	3.4
Number of introns/Mb	339	543	462
Intron GC content (%)	30.8	32.8	32.0

*Genes, exons and introns from the GLEAN gene prediction data were used in this analysis.

### Heterochromatin and recombination rate

Heterochromatic regions have been shown to display much lower rates of recombination than euchromatic regions [13,43,44]. Low recombination rates in heterochromatin have been observed in *Drosophila *and other species [13,43,44], and are often associated with accumulation of repetitive DNA. Differences in recombination rate within heterochromatic regions may differ for each chromosome based on gene densities, and/or DNA arrangement [44].

To determine whether the recombination rate is lower in the regions accumulating repetitive DNA in *Tribolium*, the genetic maps were aligned with physical maps (sequences) and the putative heterochromatic and euchromatic regions identified in each chromosome. The physical length (kb) per recombination unit (cM) was calculated for scaffolds possessing multiple markers in regions identified as putative heterochromatin or euchromatin. Due to insufficient marker densities, we could not compare recombination rates on CH1(X) and CH5. Scaffolds at the ends of chromosomes and scaffolds containing markers whose linear order on the linkage map did not agree with the order derived from the sequence data were not considered in this analysis. Thus, of 384 possible markers [2], only 275 were used in these calculations. The chi-square goodness-of-fit test was applied to the average rates of recombination in these regions. While no significant differences were detected in recombination rates between the putative heterochromatin and euchromatin on CH9, the other seven chromosomes considered (CH2, CH3, CH4, CH6, CH7, CH8 and CH10) show significantly reduced recombination rates in regions containing a high density of repetitive DNA (Table 9). Recombination in the putative heterochromatic regions on these chromosomes varies approximately 4.6-fold, from 194.8 to 893.5 kb/cM. In comparison, the rate of recombination in the putative euchromatin on these chromosomes varies only approximately 2.1-fold, from 130.2 to 245.0 kb/cM. Thus, although there are few regions in which to make valid comparisons, analysis of these regions indicates a noticeable reduction in the rate of recombination in regions containing a high density of repetitive DNA, supporting our hypothesis that these regions are heterochromatic.

**Table 9:** Recombination rate as reflected in physical size of recombination units in putative heterochromatin and euchromatin in the *Tribolium *genome assembly

Linkage group	Average physical size of a recombination unit (kb/cM)		
		
	Heterochromatin	Euchromatin	*P*
CH1*	-	-	-
CH2	Range: 721.1, 523.9, 463.9, 208.3	Range: 130.7, 153.1, 218.5	<0.01
	Average: 479.3	Average: 167.4	
CH3	Range: 322.5^†^	Range: 184.4, 226.8, 198.6, 176.4	<0.01
	Average: 322.5	Average: 196.6	
CH4	Range: 346.3, 1440.7	Range: 141.2, 200.6, 318.3	<0.01
	Average: 893.5	Average: 220.3	
CH5*	-	Range: 247.7, 320.9, 176.4, 397.2, 225.0	-
		Average: 273.4	
CH6	Range: 145.1, 244.5	Range: 191.4, 38.7	<0.01
	Average: 194.8	Average: 130.2	
CH7	Range: 440.8^†^	Range: 132.2, 257.0, 31.6, 255.8	<0.01
	Average: 440.8	Average: 169.2	
CH8	Range: 318.5, 543.2	Range: 165.5, 110.3, 98.2, 367.3	<0.01
	Average: 426.4	Average: 185.4	
CH9^‡^	Range: 195.9, 326.2, 296.0	Range: 234.3, 336.6, 164.1	-
	Average: 272.7	Average: 245.0	
CH10	Range: 241.7^†^	Range: 237.9, 127.5	<0.01
	Average: 241.7	Average: 182.7	

*Not enough genetic markers for analysis. ^†^Recombination was calculated for one scaffold that falls in heterochromatin. ^‡^No significant difference was observed in the average physical size of a recombination unit in heterochromatin versus euchromatin (*P *= 0.179).

### Abundance of repetitive DNA in *Tribolium*

The total repetitive DNA content in regions predicted to be derived from heterochromatin is greater (35.6%) than that in putative euchromatin (16.5%). This is true also when considering just TEs, which comprise 6.9% of putative heterochromatin and only 1.6% of putative euchromatin. By these criteria, the abundance of TEs in both the putative heterochromatin and euchromatin in *Tribolium *is much lower than that in *Drosophila *(15.1% in heterochromatin and 3.7% in euchromatin [28]) and *Anopheles *(60% in heterochromatin and 16% of euchromatin [29]). However, these estimates for *Trioblium *are likely to be low, since the genome assembly relied predominantly on automated methods and our search for TEs in *Tribolium *was based on homology to known TE families. Moreover, 20 Mb of the assembled genome sequence is not anchored in chromosomes and 60% of these unmapped sequences are composed of repetitive DNA (Figures 1 and 3).

### Completeness of the genome sequence and assembly

Previous estimates of the size of the *Tribolium *genome using re-association kinetics [9] or densitometric measurement of Feulgen-stained spermatids [46] are in excellent agreement at 0.2 pg or 204 Mb. However, the assembled genome sequence is only 151 Mb, a figure that increases to 160 Mb when sequencing gaps within the scaffolds are included. Thus, perhaps as much as 44 Mb of additional sequence is yet to be analyzed. Coverage of the transcribed regions of the genome appears to be quite good in that >98% of expressed sequence tags are found in the assembly [47]. Previous estimates, based on *Hpa*II-banding of chromosomes, suggest that approximately 40% of the *Tribolium *genome (81.6 Mb) is composed of heterochromatin [17]. We suggest that the intervals along the chromosomes that accumulate TEs and repetitive DNA (54.7 Mb) consist largely of heterochromatin. Even if they consist entirely of heterochromatin, there remains about 27 Mb (81.6 - 54.7) of additional heterochromatin to be analyzed. For example, the 360 bp satellite is estimated to occupy 17% of the genome [16], yet we found that only 0.3% of the genome assembly consists of this repeat element. Regions containing long tandem arrays that have been rearranged by insertion, deletion or unequal crossing-over are likely to be the most difficult to sequence or assemble, and the large number of sequencing gaps in these intervals may be due to such arrays.

## Conclusion

We identified more than 30% of the *Tribolium *genome as composed of repetitive DNA, including TEs and satellites. *Tribolium *contains a higher percentage of long satellites (>100 bp) than *Drosophila*. The distribution pattern of TEs and long satellites resemble the location of pericentric heterochromatin blocks characterized by *Hpa*II-banding in *T. castaneum*. Further analysis of these regions revealed lower gene density, lower recombination rate, and genes with longer introns than found in regions thought to be derived from euchromatin. However, given that the estimated genome size of 204 Mb is 44 Mb larger than the assembled genome sequence, there is likely more heterochromatin to be sequenced and assembled.

## Materials and methods

### Sequence files

Release 2 of the *T. castaneum *genome sequence (Tcas_2.0) and the GLEAN gene prediction files, which represent a consensus of all the *ab initio *gene predictions, were downloaded from the FTP site at the HGSC Baylor College of Medicine [47]. The euchromatin sequence of *D. melanogaster *was downloaded from FlyBase (release 4.3) [48]. The heterochromatin sequence of *D. melanogaster *was downloaded from *Drosophila *Heterochromatin Genome Project (DHGP release 3.2b) [49].

### Tandem repeat identification

Tandem repeats were identified using TRF software [19], which uses statistical criteria and dynamic programming to determine repeat units and identify tandem arrays. In this study, the alignment parameters (2, 7, 7) were used; the minimum alignment score to report a repeat was 30; and the maximum period size was 500 (the distance between corresponding characters in the alignment of tandem repeats). Perl scripts were written to eliminate redundancy and calculate the abundance and density of microsatellites, minisatellites, and satellites. We defined repeat units of 1-6 bp as microsatellites, 7-100 bp as minisatellites and >100 bp as satellites.

### *De novo *identification of repetitive DNA using RepeatScout

RepeatScout [21] was used to analyze the repetitive DNA in the *T. castaneum *and *D. melanogaster *genomes, generating repeat family libraries for each. The default parameters (seed length *l *= 15, *l *mer frequency threshold *m *= 3, repeat frequency threshold *c *= 3, alignment match score = 1, mismatch score = -1, and gap penalty = -5) were used. The minimum element length to report was 50 bp. Low-complexity repeats and tandem repeats were removed as part of the RepeatScout algorithm, using Nseg [32] and TRF [19]. Repeats having significant hits to known proteins in UniProt Release 6.0 [33] were removed from the repeat family libraries. The May 3, 2005, version of RepeatMasker [22] was used to identify repeats from each library using in the *T. castaneum *and *D. melanogaster *genomes, respectively, with default parameters. Perl scripts were written to parse the results from RepeatMasker [22] and calculate the abundance of each repeat in the *Tribolium *genome.

### Homology search for transposable elements

The identification of DNA transposons as well as non-LTR and LTR retrotransposons in the *Tribolium *genome using TEpipe will be described in detail elsewhere. In this study, we used these TE libraries to run RepeatMasker [22] on the *T. castaneum *genome assembly. Perl scripts were written to parse the results of RepeatMasker [22] and calculate the abundance of the TE in each chromosome using a cutoff length of 50 bp.

### Abundance and density calculations

The *T. castaneum *genome sequence files in Release Tcas_2.0 contain 300 kb placeholders (strings of Ns) between individual scaffolds on each chromosome build. Placeholders and sequencing gaps were excluded from our calculations of abundance and density of repetitive DNA in the assembled *Tribolium *genome. The size of the *Tribolium *genome, including placeholders and sequencing gaps, is 209,366,138 bp. Removing 48,900,000 bp of placeholder Ns yields a genome size of 160,466,138 bp, and removing 9,132,403 bp of sequencing gaps results in a genome size of 151,333,735 bp. However, when we divided the anchored genome sequence (137.7 Mb) into 0.5 Mb intervals to determine the distribution patterns of repetitive DNA, only the placeholders were eliminated to produce the best estimates of interval length.

## Abbreviations

CH, chromosome; LTR, long terminal repeat; TE, transposable element; TRF; Tandem Repeat Finder.

## Authors' contributions

SW and SB designed the analysis. SW performed all the analyses. SB, ML and RB constructed the genetic map and integrated the genetic and physical maps. SW wrote the first draft of the manuscript, which was edited by all authors, who have seen and approved the final manuscript.

## Additional data files

The following additional data are available. Additional data file 1 is a table listing amount and distribution of TEs in each chromosome of *T. castaneum*. Additional data file 2 is a text file containing the sequences of RepeatScout library repeat families (FASTA format). Additional data file 3 is a table comparing TEs in the TEpipe and RepeatScout libraries. Additional data file 4 is an Excel spreadsheet listing detailed information about each RepeatScout repeat family, for example, length, GC content, copy number in the genome, type, and percent of the genome occupied. Additional data file 5 is a figure displaying the amount of repetitive DNA in 100 kb intervals. Additional data file 6 is a table listing the putative heterochromatic regions of each chromosome in *T. castaneum*

## Supplementary Material

Additional data file 1 Amount and distribution of TEs in each chromosome of *T. castaneum*. Click here for file

Additional data file 2 Sequences of RepeatScout library repeat families (FASTA format). Click here for file

Additional data file 3 Comparison of TEs in the TEpipe and RepeatScout libraries. Click here for file

Additional data file 4 Detailed information about each RepeatScout repeat family, for example, length, GC content, copy number in the genome, type, and percent of the genome occupied. Click here for file

Additional data file 5 The amount of repetitive DNA in 100 kb intervals. Click here for file

Additional data file 6 Putative heterochromatic regions of each chromosome in *T. castaneum*. Click here for file
