# Supplementary material for: Analysis of repetitive DNA distribution patterns in the Tribolium castaneum genome
Source: Genome Biol. 2008 Mar 26;9(3):R61. doi: 10.1186/gb-2008-9-3-r61 (PMC2397513; doi:10.1186/gb-2008-9-3-r61)
Supplement: Additional data file 1 — Amount and distribution of TEs in each chromosome of T. castaneum. [file gb-2008-9-3-r61-S1.doc]

**Additional data 1.** Abundance and distribution of transposable elements found by TEpipe among the *Tribolium* chromosomes

|  | | Non-LTR | | | | LTR | | | | DNA transposon | | | | Total TE | | | |
| --- | --- | --- | --- | --- | --- | --- | --- | --- | --- | --- | --- | --- | --- | --- | --- | --- | --- |
| Linkage group | Length^a^  (Mb) | TE size  (kp) | %  of CH^b^ | Num of TE | TE  density^c^ | TE size  (kp) | %  of CH | Num of TE | TE density  /Mb | TE size  (kp) | % of CH | Num of TE | TE density | TE size  (kp) | %  of CH | Num  of TE | TE  density |
| CH1(X) | 7.0 | 46 | 0.66 | 212 | 30.2 | 49 | 0.71 | 185 | 26.4 | 50 | 0.72 | 274 | 39.1 | 147 | 2.10 | 671 | 95.7 |
| CH2 | 14.0 | 106 | 0.76 | 526 | 37.5 | 82 | 0.59 | 499 | 35.6 | 214 | 1.53 | 1396 | 99.6 | 403 | 2.88 | 2421 | 172.8 |
| CH3 | 28.4 | 363 | 1.28 | 1579 | 55.5 | 271 | 0.95 | 1608 | 56.5 | 785 | 2.76 | 4816 | 169.3 | 1420 | 4.99 | 8003 | 281.3 |
| CH4 | 11.5 | 94 | 0.82 | 442 | 38.3 | 154 | 1.34 | 465 | 40.3 | 161 | 1.40 | 1043 | 90.4 | 411 | 3.56 | 1950 | 169.0 |
| CH5 | 13.6 | 90 | 0.67 | 467 | 34.4 | 60 | 0.45 | 439 | 32.3 | 159 | 1.17 | 1041 | 76.6 | 310 | 2.28 | 1947 | 143.3 |
| CH6 | 8.2 | 115 | 1.40 | 478 | 58.1 | 108 | 1.32 | 444 | 54.0 | 218 | 2.65 | 1236 | 150.2 | 442 | 5.38 | 2158 | 262.2 |
| CH7 | 14.0 | 86 | 0.61 | 409 | 29.1 | 148 | 1.06 | 573 | 40.7 | 193 | 1.38 | 1215 | 86.3 | 429 | 3.05 | 2197 | 156.1 |
| CH8 | 12.7 | 159 | 1.25 | 753 | 59.0 | 130 | 1.03 | 643 | 50.4 | 282 | 2.21 | 1722 | 134.9 | 571 | 4.48 | 3118 | 244.3 |
| CH9 | 14.6 | 219 | 1.51 | 900 | 61.8 | 123 | 0.85 | 778 | 53.4 | 305 | 2.09 | 1931 | 132.6 | 648 | 4.45 | 3609 | 247.7 |
| LG10 | 6.7 | 87 | 1.30 | 404 | 60.3 | 77 | 1.16 | 314 | 46.8 | 208 | 3.11 | 1154 | 172.2 | 373 | 5.57 | 1872 | 279.3 |
| unmapped | 20.4 | 1588 | 7.79 | 4943 | 242.3 | 1377 | 6.75 | 3751 | 183.9 | 675 | 3.31 | 3497 | 171.4 | 3641 | 17.85 | 12191 | 597.6 |
| Total | 151.3 | 2958 | 1.96 | 11113 | 73.4 | 2585 | 1.71 | 9699 | 64.1 | 3254 | 2.15 | 19325 | 127.7 | 8798 | 5.81 | 40137 | 265.2 |

^a^Chromosomelength does not include 300-kb placeholders and sequencing gaps.

^b^Transposable element libraries were produced by TEpipe. RepeatMasker was used to determine the % of each CH composed of TEs. Length cutoff = 50 bp.

^c^TE density = Total number of TE loci/ chromosome (Mb)
