# Supplementary material for: Analysis of repetitive DNA distribution patterns in the Tribolium castaneum genome
Source: Genome Biol. 2008 Mar 26;9(3):R61. doi: 10.1186/gb-2008-9-3-r61 (PMC2397513; doi:10.1186/gb-2008-9-3-r61)
Supplement: Additional data file 3 — Comparison of TEs in the TEpipe and RepeatScout libraries. [file gb-2008-9-3-r61-S3.doc]

**Additional data 3.** Comparison of transposable elements

in the TEpipe and RepeatScout libraries

| TE Class | Proportion of RepeatScout library (%) | TEpipe elements  identified by RepeatScout (%) |
| --- | --- | --- |
| Non-LTR | 13.0 | 76.4 |
| LTR | 12.0 | 63.6 |
| DNA transposons | 3.4 | 65.3 |
