# Supplementary material for: Analysis of repetitive DNA distribution patterns in the Tribolium castaneum genome
Source: Genome Biol. 2008 Mar 26;9(3):R61. doi: 10.1186/gb-2008-9-3-r61 (PMC2397513; doi:10.1186/gb-2008-9-3-r61)
Supplement: Additional data file 6 — Putative heterochromatic regions of each chromosome in T. castaneum. [file gb-2008-9-3-r61-S6.doc]

Additional data 6**.** The putative heterochromatic regions on each chromosome in *Tribolium castaneum*

| Chromosome | Length  (Mb) | Position of heterochromatin  (Mb) | Amount of heterochromatin  (Mb) | Percent of CH  (%) |
| --- | --- | --- | --- | --- |
| CH1 (X) | 7.3 | 5.0–7.2 | 2.2 | 30.1 |
| CH2 | 14.5 | 0–5.0, 5.5–6.0, 7.5–8.0 | 6.0 | 41.4 |
| CH3 | 30.1 | 11.0–17.5, 18.0–21.0, 22.0–23.0,  23.5–24.5, 25.5–26.0 | 12.0 | 39.9 |
| CH4 | 12.1 | 3.0–3.5, 8.5–12.0 | 4.0 | 33.1 |
| CH5 | 14.1 | 0–4.5, 9.5–10.5 | 5.5 | 39.0 |
| CH6 | 9.0 | 5.0–5.5, 6.0–9.0 | 3.5 | 38.9 |
| CH7 | 14.6 | 0–0.5, 5.0–5.5, 6.5–7.5,  12.5–14.5 | 4.0 | 27.4 |
| CH8 | 13.5 | 0–2.0, 2.5–5.5, 6.0–7.0 | 6.0 | 44.4 |
| CH9 | 15.5 | 0.5–1.0, 1.5–4.0, 5.0–10.0 | 8.0 | 51.6 |
| CH10 | 7.1 | 0–3.0, 5.0–5.5 | 3.5 | 49.3 |
| Total | 137.8 |  | 54.7 | 39.7 |
